# Revealing the Control Mechanisms of pH on the Solution Properties of Chitin via Single-Molecule Studies

**DOI:** 10.3390/molecules28196769

**Published:** 2023-09-22

**Authors:** Song Zhang, Miao Yu, Guoqiang Zhang, Guanmei He, Yunxu Ji, Juan Dong, Huayan Zheng, Lu Qian

**Affiliations:** 1 Department of Food Science and Engineering, Moutai Institute, Renhuai 564502, China; songzhang93@126.com (S.Z.); gq198615@163.com (G.Z.); 17685204473@163.com (G.H.); m18936395582@163.com (Y.J.); 18586724865@163.com (J.D.); 2 School of Mechanical Engineering, Sichuan University, Chengdu 610065, China; miaoyu@scu.edu.cn; 3 School of Materials Science and Engineering, South China University of Technology, Guangzhou 510641, China

**Keywords:** chitin, single-molecule force spectroscopy, molecular dynamic simulations, non-covalent interactions, elasticity

## Abstract

Chitin is one of the most common polysaccharides and is abundant in the cell walls of fungi and the shells of insects and aquatic organisms as a skeleton. The mechanism of how chitin responds to pH is essential to the precise control of brewing and the design of smart chitin materials. However, this molecular mechanism remains a mystery. Results from single-molecule studies, including single-molecule force spectroscopy (SMFS), AFM imaging, and molecular dynamic (MD) simulations, have shown that the mechanical and conformational behaviors of chitin molecules show surprising pH responsiveness. This can be compared with how, in natural aqueous solutions, chitin tends to form a more relaxed spreading conformation and show considerable elasticity under low stretching forces in acidic conditions. However, its molecular chain collapses into a rigid globule in alkaline solutions. The results show that the chain state of chitin can be regulated by the proportions of inter- and intramolecular H-bonds, which are determined via the number of water bridges on the chain under different pH values. This basic study may be helpful for understanding the cellular activities of fungi under pH stress and the design of chitin-based drug carriers.

## 1. Introduction

**Figure 1:**
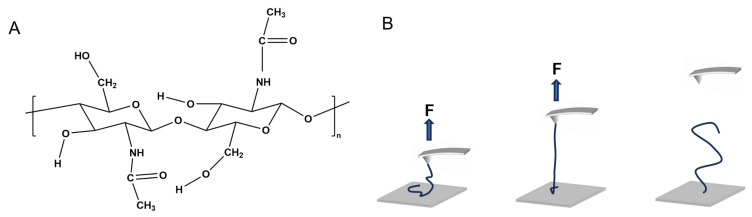
(**A**) The chemical structure of chitin. (**B**) A schematic drawing of the working principle of SMFS.

Chitin, as the second-most abundant biomacromolecule after cellulose, is widely distributed in biological species, including all fungal species and many insects and invertebrate aquatic organisms. Similar to cellulose, chitin is a structural polysaccharide composed of β-(1-4)-linked N-acetylglucosamine (GlcNAc) repeating units (Figure 1A). The only structural difference between these two polymers is the alkyl group side chain of the saccharide ring [1]. In fungi, chitin is located in the innermost layer of the cell wall, which is generally close to the phospholipid bilayer. The mass fraction of chitin in the cell wall of brewing beneficial fungi (yeasts) can be up to 15%. The inner chitin layer ensures the stability of the cell wall and plays a key role in functions such as protecting fungi from external stress and embedding important proteins in the cell wall and membrane. As an important type of biomaterial, chitin hydrogels have been widely used as wound dressings because of their biocompatibility and ability to provide a moist environment for wound healing [2,3,4,5]. In addition, the use of chitin-based hydrogels as drug carriers has aroused a great deal of attention [6,7,8,9].

The biological properties of chitin are closely related to the environment in which it is found. In particular, an environment’s pH value will affect the distribution of chitin around the cell membrane, which plays an important role in regulating the proliferation and growth of yeast [1,10,11,12,13,14,15]. In one report, chitin was found to be abundantly distributed on the external cell wall surface during the isotropic growth of a daughter cell [16,17]. Moreover, the intracell liquids were proven to be acidic (pH = 4–6) in this period [18]. The growth rate of the cells and the content of chitin in the cell wall were both clearly increasing under acidic conditions, which may be related to the morphology of chitin [19]. In addition, pH is also deeply involved in the properties of chitin materials. When chitin is used as a drug carrier, the drug release efficiency at an acidic pH is better than that at a neutral pH [2,15,20,21]. Even though the functions of fungi and the performance of chitin-based hydrogel drugs are deeply related to pH values, the mechanisms of the mechanical and conformational behaviors of chitin in aqueous environments at different pH values remain unclear at the molecular level [2,22,23]. Furthermore, it is difficult to precisely analyze the cell properties of fungi under pH stress and the interactions between chitin-containing drugs and the environment.

In recent decades, single-molecule force spectroscopy (SMFS) has shown its powerful ability in the investigation of the mechanical, conformational, and structural features of both biomacromolecules and synthetic polymers [24,25,26,27,28,29,30,31,32,33,34,35,36]. One of the most prominent features of SMFS is that measurements can be carried out in various conditions (in a liquid, in air, or in a vacuum) [37].

In this study, SMFS was combined with AFM imaging and molecular dynamic (MD) simulations [38] to investigate the single-molecule behaviors of chitin in aqueous environments with different pH levels. The results show that both molecular conformation and elasticity can be deeply affected by pH. Under acidic conditions, chitin shows a spreading free-chain conformation and an outstanding elasticity under low forces, while in alkaline solutions, chitin collapses into a hydrophobic globule and assumes a “stiff” mechanical character. It is believed that the strength of charge repulsion along the chain direction, which has a large influence on the number of water bridges between the adjacent saccharide rings of chitin and the assignment of H-bonds on chitin, is the key factor determining the state of a chitin molecule but shows no obvious influence on the elasticity of chitin.

## 2. Results and Discussion

### 2.1. Molecular Mechanical Properties of Chitin in DI Water

**Figure 2:**
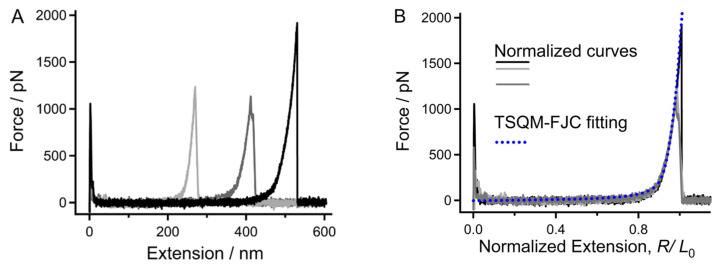
Typical F-E curves of chitin obtained in DI water. (**A**) Original F-E curve. (**B**) Normalized effect of those shown in (**A**), and the fitting result obtained using the TSQM-FJC model.

To facilitate the comparison of results from SMFS performed in aqueous conditions, we first investigated the single-molecule mechanical properties of chitin in deionized (DI) water. Figure 2A shows the typical force–extension (F-E) curves of single chitin chains measured via SMFS. The narrow force peak at the initial part of each curve corresponds to the adhesive effect when the AFM tip moves away from the substrate [39]. As the molecular chain is stretched from the free state to the extended state, the second force peak appears and rises gradually with the extension. Finally, the force reaches a maximum and drops to zero abruptly, indicating that the molecular bridge between AFM tip and the substrate has been broken [40,41]. Due to the variation in the molecular weight of each stretched chitin chain, the locations of the second force peak of the curves are different. To compare the elasticity directly, the F-E curves need to be further normalized.

The molecular elasticity of a polymer chain in an environment is provided by enthalpy and entropy. For chitin, a polymer linked by saccharide ring units, the elasticity along the chain direction under high force should be dominated by enthalpic elasticity from the ring strain. That is, the single-molecule elasticity of chitin can hardly be affected by environments under high forces. In general, the strength of strong non-covalent interactions (such intrachain H-bonds and π-π interactions) is not more than 1500 pN [29]. This means that a force region higher than 1500 pN reflects the inherent elasticity of a molecular chain, and the F-E curves of one polymer obtained in different environments should show a similar elasticity when the force is greater than 1500 pN. Therefore, the F-E curves shown in Figure 2A were normalized via their extension at 1500 pN, which is close to the strength of covalent bonds [42]. As shown in Figure 2B, the normalized experimental F-E curves can be overlapped neatly, suggesting that they have the same elasticity and should all be the stretching result of single chitin chains.

Chitin is a polysaccharide that is linked by 1,4-glycosidic bonds. The inherent elasticity of this kind of polysaccharide, in addition to others such as cellulose and amylose, was determined by Cui et al. through quantum-mechanical (QM) calculations [37]. Considering that there are many polar groups on its sidechain, one can conclude that water molecules can bind with chitin through H-bonds. By introducing the QM calculation results into the freely jointed chain (FJC) model (Equation (1)), which considered the kinetics of noncovalent interactions with two states, the new model (the TSQM-FJC model, Equation (2)) can be used to describe the elasticity of chitin under aqueous conditions [43]. The elastic modulus used in the QM-FJC model has been calculated previously based on the backbone of cellulose [44].

(1) $$Z_{\mathrm{FJC}}=\frac{R}{L_{0}}=\left(L\left[F\right]L_{0}\right)\left\{\coth\left[\frac{Fl_{k}}{k_{B}T}\right]-\left(k_{B}T\right)/\left(Fl_{k}\right)\right\}$$

In Equation (1), Z_FJC_ is the normalized extension of a chitin chain, and *l*_k_ is the length of the Kuhn segment (0.514 nm for chitin); the stretching force F is the only free parameter.

(2) $$\begin{array}{c} z_{\mathrm{TS}}=\frac{R_{\mathrm{TS}}}{L_{0}}=\frac{L_{\mathrm{TS}}.z_{\mathrm{FJC}}}{Nl_{u}}=\left[\frac{l_{f}}{e^{\frac{-\Delta G+F\Delta L}{k_{B}T}}+1}+\frac{l_{u}}{e^{\frac{\Delta G-F\Delta L}{k_{B}T}}+1}\right].z_{\mathrm{FJC}}/l_{u} \end{array}\;$$

**Figure 3:**
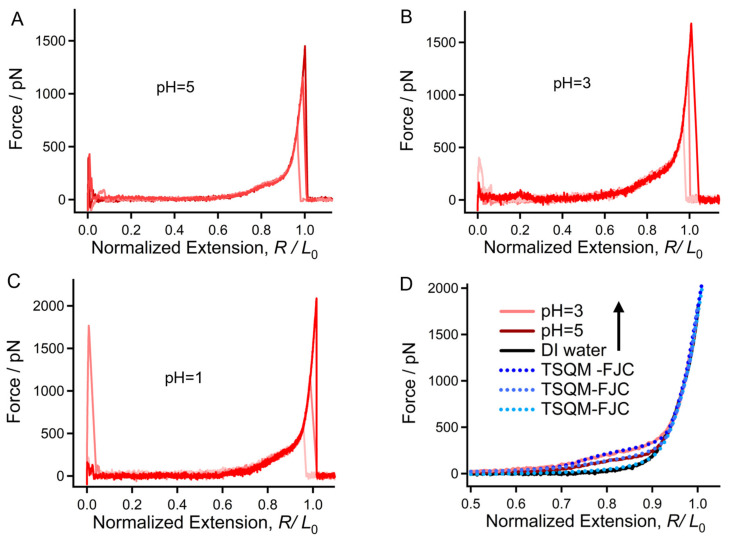
Typical normalized F-E curves of chitin obtained in HCl aqueous solutions of pH = 5 (**A**), pH = 3 (**B**), and pH = 1 (**C**). (**D**) Direct comparison of the F-E curves of chitin obtained in acidic conditions and the fitting result obtained using the TSQM-FJC model.

In Equation (2), the value of l_u_ is the length of a saccharide ring of chitin in a free state (0.45 nm), and *l*_f_ is its length under a high force (0.54 nm). Because ΔL = *l*_u_ − *l*_f_, there are only two free parameters, ΔG and l_f_. One can notice that the single-molecule elasticity of chitin obtained in DI water can be fitted adequately by the TSQM-FJC model with a given set of parameters, that is, ΔG = 1.50 k_B_T/unit = 3.75 kJ/(mol·unit), ΔL = 0.09 nm, *l*_f_ = 0.54 nm, and *l*_u_ = 0.45 nm (Figure 3B). These results indicate that the average strength of water bridges between adjacent saccharide units is 3.75 kJ/(mol·unit). Note that the ΔG of chitin in DI water is much lower than that of amylose (5.1 k_B_T/unit = 12.75 kJ/(mol·unit)) [45], a water-soluble polysaccharide that shares the same backbone elasticity with cellulose [44]. Considering that these two polysaccharides share nearly the same backbone structure and that chitin possesses even more polar groups than amylose, we suppose that a single chitin chain is relatively weak when hydrated in DI water.

### 2.2. Surprising Enhancement of Single-Molecule Elasticity Due to Strong Hydratability in Acid

To the best of our knowledge, although many studies focus on the influence of pH on the expression of fungi and the basic performance of chitin materials and other glucose-derivatives (such as chitosan), the corresponding molecular mechanisms remain largely unknown due to the complexity of the systems [19,46]. Figure 3A–C show the F-E curves of chitin obtained in aqueous HCl solutions with different pH values. Interestingly, there is a noticeable shoulder-like force plateau on each curve at the low-force region (from about 150–250 pN), which is determined by the entropy of the elasticity of a polymer chain. When compared, the experimental F-E curves obtained in acidic conditions can be adequately overlapped with those obtained in DI water at the high-force region (over 500 pN), while showing a significant deviation at lower force levels. Compared to the F-E curve obtained in DI water, a gentle shoulder-like force plateau appears at about 150 pN, indicating that more bound water molecules are serving as bridges linking the adjacent saccharide units. The height of the shoulder-like force plateau rises to about 250 pN when the pH decreases to 3 and remains steady under lower pH conditions. The control experiments carried out in NaCl solutions with a concentration gradient (not exceeding 0.1 M) demonstrate that the presence of Cl^-^ can hardly influence the elasticity of chitin in water. Therefore, we have reason to believe that the shoulder-like force plateau of the F-E curves obtained in acidic conditions is mainly determined by H^+^.

Herein, the TSQM-FJC model was used to quantitatively study the influence of H^+^ on the mechanical properties of chitin. It was found that the F-E curves obtained at pH = 3 (or pH = 1) can be fitted well by the model when ΔG = 5.53 k_B_T/unit = 13.83 kJ/(mol·unit). In addition, the energy difference between the pH = 3 and DI water was calculated to be 9.08 kJ/(mol·unit), which is remarkable for a natural or synthetic polymer in different aqueous environments [46]. In acid, many H^+^ can bind to water molecules to form H_3_O^+^ and further act as bridges between the adjacent sugar units of a molecular chain [47]. Compared to a common water bridge, the repulsive effect of the charges among the bound H_3_O^+^ aids the spreading of a molecular chain. Accordingly, more water bridges form on a chitin chain. When stretched under an external force, the bridges linked by H-bonds will be broken with the extension. The more water bridges, the larger the ΔG required, causing a higher shoulder-like force plateau in the F-E curve. Because the number of surviving water bridges is very limited under higher forces (over 500 pN), single chitin molecules being stretched in acidic conditions show similar elasticity in this force region. It is interesting to further discuss the conformational behavior of chitin under relatively mild conditions. The typical F-E curve of chitin obtained at pH = 5 shows obvious deviation from that obtained in DI water (Figure 3D in the manuscript). The ΔG required to stretch a chitin chain between the two states was calculated to be 8.53 kJ/(mol·unit) via TSQM-FJC fitting (Figure 3D), which is about 62% of the ΔG between pH = 3 and DI water (13.83 kJ/(mol·unit)). Considering that the value of ΔG shows a positive correlation with the amount of chain spreading under acidic conditions, we can make a rough prediction that the spreading degree of chitin at pH = 5 is 62% that of its final state under acidic conditions (pH ≤ 3). The pH-induced single-molecule mechanical transition at low-force regions may aid in the design of intelligent elastic chitin hydrogels.

### 2.3. Mechanical Properties of Chitin Chains in Alkaline Environments

**Figure 4:**
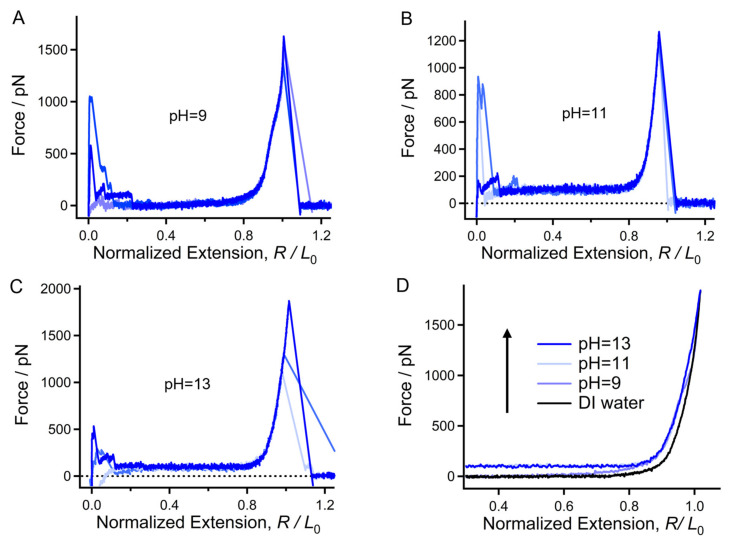
(**A**–**C**) Typical normalized F-E curves of chitin obtained in NaOH solutions with different pH values. (**D**) Direct comparison of the typical F-E curves shown in (**A**–**C**).

Previous studies have shown that alkali/urea systems aid in the dissolution of chitin and the design of chitin hydrogels [48]. However, there is a lack of molecular-level evidence regarding whether OH^−^ can improve the hydratability of chitin. The above SMFS studies conducted in acids indicate that hydratability can be reflected by mechanical variations. Figure 4 shows the normalized F-E curves of chitin obtained in NaOH solutions with different pH values. The elasticity of a polymer chain is provided by both covalent and noncovalent interactions. When the external force is large enough, the elasticity is mainly determined by covalent bonds [42,49]. Therefore, the typical F-E curves obtained in pH = 9, 11, 13 and DI water can be overlapped well under high forces (over 1500 pN) since they correspond to the stretching of a single chitin molecule (Figure 4D). Notably, the curves show significant deviation below 1500 pN. In this force region, the curve obtained at pH = 9 is above that obtained in DI water and shows a sharp shoulder-like force plateau at about 700 pN (Figure 4D). The force deviation becomes more obvious when the pH increases to 11 and remains steady under higher-pH conditions. The noncovalent interactions of chitin chains are dominated by intrachain and intermolecular H-bonds, which compete with each other and bring about low forces and a higher force region, respectively [45]. Therefore, when the number of intermolecular hydrogen bonds is dominant (i.e., the situation in acid), a shoulder-like force plateau appears in the low-force region (150–250 pN). The much higher shoulder-like force plateau (about 700 pN) at pH = 9 indicates that the intrachain nonbonding interaction is strengthened compared to that in acidic environments and DI water. These results demonstrate that the hydratability of the chitin molecule may be weakened by the presence of OH^−^.

### 2.4. Origin of the Mechanical Deviation of Chitin in Acid, DI Water, and Alkali Medium in Relation to the Aspect of Molecular Morphology

**Figure 5:**
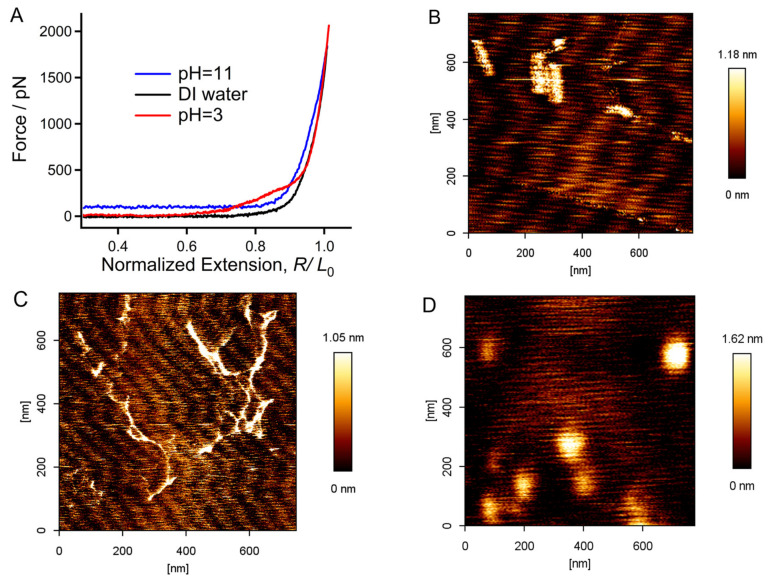
(**A**) Direct comparison of the F-E curves of chitin obtained at pH = 3, in DI water, and at pH = 11. (**B**–**D**) Molecular morphology of chitin obtained in DI water, at pH = 3, and at pH = 11, respectively.

The F-E curves of chitin obtained at pH = 3, in DI water, and at pH = 11 were compared for a further analysis (Figure 5A). It is obvious that the curves show distinct divergence below 1500 pN. Compared to that in DI water, the stretching force of the chitin chain increases in both the acidic and alkaline environments. The difference is that the alkaline condition shows a greater influence in the higher force region, indicating that the molecule has become “stiff”, while the influence in the lower force region is more prominent under acidic conditions, which reflects the high elasticity of chitin under these conditions. The long stable force plateau that appears at about 100 pN before elastic stretching in the F-E curves obtained at pH = 11 (or 13) may correspond to the elongation of the chitin molecule in a hydrophobic globule state [37,50]. AFM imaging experiments of the extremely dilute chitin solution were carried out for confirmation. As shown in Figure 5B, the chitin molecules tended to form semi-collapsed structures in DI water, and this is consistent with their low ΔG (3.75 kJ/(mol·unit)) determined via TSQM-FJC fitting. Surprisingly, the molecules transform into spreading free chains when the pH was equal to 3, indicating that the molecules can be sufficiently hydrated in this environment, where they show remarkable hydration energy (ΔG = 13.83 kJ/(mol·unit)). Accumulated, collapsed globules were found to be the main morphology of chitin when the pH was increased to 11. Moreover, the thickness of chitin on the substrate increases with an increasing pH. The AFM imaging results confirmed that the long force plateaus at about 100 pN shown in Figure 4B,C were caused by the hydrophobic effect of chitin in alkaline conditions.

### 2.5. Analysis of the Single-Molecule Physical Properties of Chitin via MD Simulations

In order to investigate the kinetic principle of a chitin chain in environments with different pH levels, MD simulations were carried out in HCl solution (pH = 3), pure water, and NaOH solution (pH = 11). Typical pictures from the simulation animations at 20 ns show that the chitin molecules adopt a spreading conformation in acid, a relatively collapsed conformation in water, and a more collapsed globule conformation in alkaline conditions. These simulation results remarkably match the AFM-imaging results. The dynamic radius of gyration (R_g_) is a parameter used to characterize molecular size that can reflect the microscopic properties of molecular chains in solution [51]. R_g_ can be written in the following form:

(3) $$R_{g}={\left(\frac{\sum_{i}{\left|\left|r_{i}\right|\right|}^{2}m_{i}}{\sum_{i}m_{i}}\right)}^{1/2}$$

**Figure 6:**
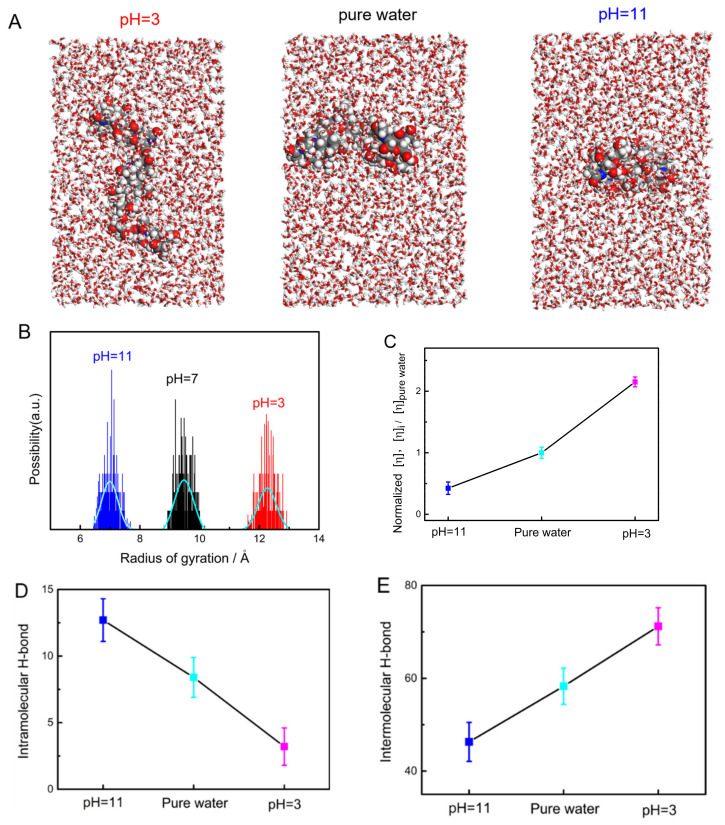
MD simulation results of chitin in aqueous solutions with different pH levels. (**A**) The typical molecular conformation of chitin under the tested conditions. (**B**) The R_g_ of a chitin chain under different conditions (blue: pH = 11, black: pure water, and red: pH = 3). (**C**–**E**) The normalized $\left[\eta \right]$ and the total number of intramolecular and intermolecular H-bonds of a chitin chain under different conditions, respectively (blue: pH = 11, cyan: pure water, and pink: pH = 3).

Above, |$m_{i}$| is the mass of the chitin chain at point i, and |$r_{i}$| is the vector from point i to the center of mass of the chitin chain. The calculated R_g_ in this study is based on the MD results from 0–20 ns. As shown in Figure 6B, the $R_{g}$ values of a chitin chain are 9.48 ± 0.02 Å in pure water, 7 ± 0.02 Å in NaOH solution at pH = 11, and 12.27 ± 0.03 Å in HCl solution at pH = 3. The ratio of the R_g_ value at pH = 3 to that in pure water is 129%, and the ratio for this value at pH = 11 to that in pure water is 74%. Because a larger R_g_ value corresponds to a more intense spreading conformation, the ratios can intuitively reflect the conformation behavior of chitin in solutions with varying pH. This result is consistent with the tendency of the end–end distance of chitin in solutions with varying pH (Figure S10). This means that chitin shows a more relaxed conformation in acid and a comparatively more constrained (or collapsed) conformation in pure water. The simulation results are consistent with the results from the SMFS and AFM imaging.

Based on the value of $R_{g}$, the characteristic viscosity of a polymer chain can be obtained from Equation (4)

(4) $$\left[\eta \right]=\frac{\frac{10{\pi N}_{A}}{3}\left(R_{g}/\rho^{3}\right)}{M}$$

where $\left[\eta \right]$ is the characteristic viscosity, which can reflect the viscous property of an extremely dilute polymer solution. ρ is the density of the cell (0.991 kg/L for the water/chitin solution, 0.989 kg/L for the NaOH/chitin aqueous solution at pH = 11, and 0.983 kg/L for the HCl/chitin aqueous solution at pH = 3) obtained via MD simulations using the NPT model. M is the molecular weight of the chitin chain with five repeating units. Figure 6C shows the calculated normalized $\left[\eta \right]$ of a chitin chain under each condition, where the $\left[\eta \right]$ in pure water was normalized to 1 as a standard. It is clear that the $\left[\eta \right]$ in the alkali environment is relatively low (0.424 times that in pure water), while the value under the acidic condition suddenly rises to 2.151 times that in pure water. As the $\left[\eta \right]$ is an important parameter that can reflect the viscosity and adhesion properties of polymers in solution, this result may aid in the design of novel chitin drugs [52,53].

### 2.6. The Intramolecular and Intermolecular H-Bonds of a Chitin Chain in Solution

As mentioned in the SMFS section, intramolecular and intermolecular H-bonds govern the noncovalent interactions of a chitin molecule in solution. In this section of our study, the ratio of the two types of H-bonds in each condition was analyzed based on the MD simulation results. As shown in Figure 6D,E, the ratio of intramolecular to intermolecular H-bonds on a chitin molecule shows a reverse trend between acidic and alkaline solutions. The number of intramolecular H-bonds increases with pH, while that of intermolecular H-bonds decreases with pH. According to the result, the ratios of intermolecular H-bonds/intramolecular H-bonds of chitin were calculated to be 24:1, 7:1, and 4:1 for pH = 3, pure water, and pH = 11, respectively. The high proportion of intermolecular H-bonds of chitin in acid agrees well with the large ΔG from TSQM-FJC fitting and the huge difference of the end–end distance of chitin under pH = 3 and 11 (Figure S10). This result is also a good explanation of why a chitin chain shows an unfolded free conformation and a large $\left[\eta \right]$ value in acid (pH = 3). It is believed that the high ratio of intramolecular H-bonds is the direct driving force causing the molecular chain to collapse into a relatively hydrophobic globule conformation.

## 3. Materials and Methods

### 3.1. Materials and Chemicals

Chitin powder was purchased from Aladdin Biochemical technology Corp. (CAS No. 1398-61-4, Shanghai, China). The water mentioned in this study is deionized (DI) water (>18 MΩ·cm). Hydrochloric acid (1 mol/L), NaOH, and other analytically pure chemicals were purchased from Sigma-Aldrich Corp (St. Louis, MO, USA).

### 3.2. Details of SMFS

Chitin was dissolved in ionic liquid (at 23 °C with agitation concussion for 1 h) to obtain a very dilute solution (10 mg/L) [54]. Then, 20 μL of the solution was poured onto a clean quartz slide for 30 min. The sample was rinsed with an ample amount of environmental liquid for SMFS experiments (DI water, HCl, or NaOH aqueous solutions with different pH levels) and air-dried before being mounted on the AFM (MFP-3D, Asylum Research). Subsequently, a drop of environmental liquid was placed between the V-shaped Si_3_N_4_ AFM cantilever (Bruker Corp., Billerica, MA, USA) and the chitin sample. During the SMFS experiment, there was a molecular bridge between the AFM tip and the substrate (Figure 1B). When the AFM tip was attached to the single molecule chitin sample under a large loading force (4 nN in this study), the polar atoms of chitin (N and O) may link with those of the Si_3_N_4_ AFM tip (N atoms) through covalent bonds. At the same time, similar links may form between chitin and the hydroxylating polar substrate [37]. Therefore, the molecular bridge can gain the strength of covalent bonds (about 1500 pN). The molecular chain is stretched under an external force, and the force increases progressively at the elastic elongation region. When the molecular chain is sufficiently stretched, the molecular bridge breaks due to the high restoring force. The relationship between the extension distance and stretching force was recorded during experiment and subsequently converted into force–extension (F-E) curves. The data were collected during SMFS measurements and then converted into force–extension (F-E) curves. The spring constant of AFM cantilever obtained via thermos excitation ranged from 30 to 50 pN/nm. The stretching velocity was 2.0 μm/s. Three independent SMFS experiments were carried out in each condition to obtain more than 100 effective F-E curves. The raw SMFS F-E curves were analyzed using Igor Pro [55].

### 3.3. Details of AFM Imaging

All imaging experiments were carried out on the AFM mentioned above at RT and the standard atmospheric pressure. In order to present the molecular morphology as accurately as possible, highly oriented pyrolytic graphite (HOPG, Bruker Corp., Billerica, MA, USA) was used as the substrate for the physically adsorbed chitin solution. A total of 10 mL of environmental solution was poured between the HOPG surface and the AFM tip (SNL-10, Bruker Corp., Billerica, MA, USA). Tapping mode in solution was used in AFM imaging experiments. The spring constant of the AFM cantilever was ~0.35 N/m, and the cantilever resonance frequency was 28–35 KHz according to the composition of solution, which can influence the surface tension between the AFM tip surface and solution. The applied line rate in this study was relatively slow (0.8 Hz) to ensure the quality of AFM images. The images were analyzed using NanoScope Analysis 1.5 software [56]. No other processing was conducted on the images except flattening. Both SMFS and imaging experiments were carried out at room temperature (RT, 298 k).

### 3.4. Details of MD Simulations

All MD simulations of single-molecule chitin in different solutions were carried out using Material Studio 2020 software package. An amorphous cell that contained a chitin chain (5 repeating units and 10 saccharide rings in total), 1200 water molecules, and other components (HCl or NaOH) of a certain proportion was constructed. The size of the cell (about 30 Å × 30 Å × 50 Å) depended on the van der Waals radius of the system. The electrostatic and van der Waals interactions employed were Ewald and Atom-based. After the model had been constructed, the cell was geometrically optimized at an ultra-fine quality before analyzing dynamics. Subsequently, an equalization treatment was performed under NPT model for 5 ns to obtain the density (ρ) of the cell. After that, dynamic simulations under NVT were performed for 20 ns for data collection. All dynamic simulations were carried out at RT. The selected thermostat was Andersen. The number of hydrogen bonds (H-bonds) corresponds to the statistical results based on the dynamic simulation during the data collection step. COMPASSII was the chosen forcefield for all cases.

## 4. Conclusions

In summary, the pH sensitivity of chitin in aqueous solutions was systematically investigated at the single-molecule level in this study through SMFS, AFM imaging, and MD simulations. The results reveal that the pH value, especially the critical pH values (3 and 11), can profoundly influence both the elasticity and the molecular conformation of a chitin chain. Under acidic conditions, benefiting from the high ratio of intermolecular H-bonds, chitin exists in a spreading free chain conformation and shows dominant elasticity under low forces. Moreover, the characteristic viscosity $\left[\eta \right]$ of chitin also significantly increases with the acidity. Chitin possesses a high ratio of intramolecular H-bonds in alkaline solutions (pH ≥ 11), helping the chain to form a hydrophobic molecular globule and possess a low $\left[\eta \right]$. This basic research may not only be helpful for the design of intelligent chitin drugs and chitin–polymer hydrogels of different natures for tissue-engineering applications and the 3D printing of biomedical materials [57,58] but also provide an opportunity to investigate the properties of the cell walls of fungi under pH stress.

## Data Availability

All the relevant data used in this study have been provided in the form of figures and tables in the published article, and all data provided in the present manuscript are available to whom they may concern.

## Supplementary Materials

The following supporting information can be downloaded at: https://www.mdpi.com/article/10.3390/molecules28196769/s1. Figure S1: Typical original F-E curves of chitin obtained at pH = 5; Figure S2: Typical original F-E curves of chitin obtained at pH = 3; Figure S3: Typical original F-E curves of chitin obtained at pH = 1; Figure S4: Typical original F-E curves of chitin obtained at pH = 9; Figure S5: Typical original F-E curves of chitin obtained at pH = 11; Figure S6: Typical original F-E curves of chitin obtained at pH = 13; Figure S7: Fitting results of TSQM-FJC and TSQM-FRC models on the F-E curves of chitin obtained in DI water. Obvious deviations can be noticed between the theoretical curves and the experiment curve; Figure S8: The area difference of chitin in alkaline solutions compared to that in DI water; Figure S9: MFJC model fitting on the F-E curves of chitin at pH = 3 and 11; Figure S10: Statistical result of the end-end distance of chitin at pH = 3, pure water and pH = 11. Reference [59] is cited in the supplementary materials.

## Sample Availability

Samples of the compounds (including chitin, hydrochloric acid and NaOH) are available from the authors.
