# Supplementary material for: Revealing the Control Mechanisms of pH on the Solution Properties of Chitin via Single-Molecule Studies
Source: Molecules. 2023 Sep 22;28(19):6769. doi: 10.3390/molecules28196769 (PMC10574145; doi:10.3390/molecules28196769)
Supplement: Supplementary file 1 [file molecules-28-06769-s001.zip › molecules-2611866-supplementary.pdf]

Supporting Information

Revealing the control mechanisms of pH on the solution properties of chitin by single-molecule studies

Song Zhang[1], Miao Yu[3], Guoqiang Zhang[1], Guanmei He[1], Yunxu Ji[1], Juan Dong[1], Huayan Zheng[1,*] and Lu Qian[2,*]

[1]Department of Food Science and Engineering, Moutai Institute, Renhuai 564502, Guizhou, P.R. China.

[2]School of Materials Science and Engineering, South China University of Technology, Guangzhou 510641, Guangdong, China.

[3]School of Mechanical Engineering, Sichuan University, Chengdu 610065, China.

*Correspondence: zhenghuayan@mtxy.edu.cn (H.Z.); luqian1721@126.com (L.Q.).

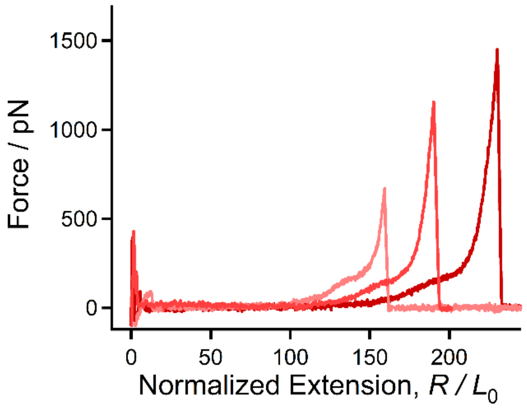

Figure S1 Typical original F-E curves of chitin obtained in pH=5.

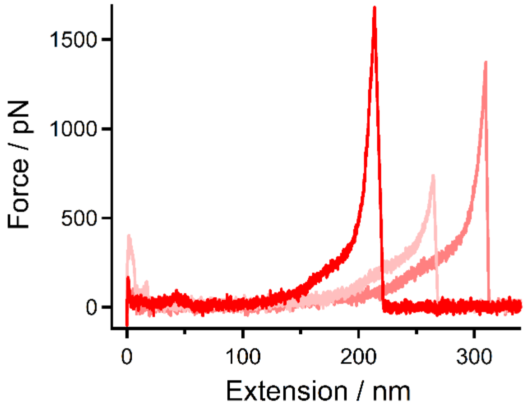

Figure S2 Typical original F-E curves of chitin obtained in pH=3.

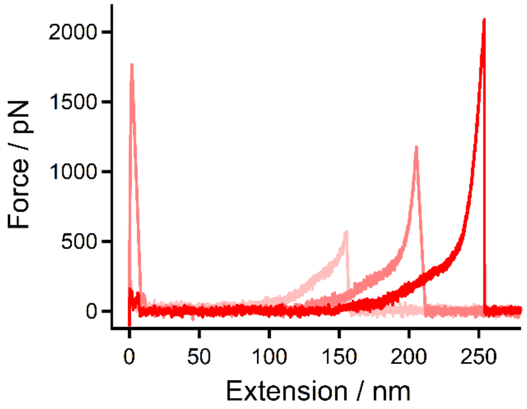

Figure S3 Typical original F-E curves of chitin obtained in pH=1.

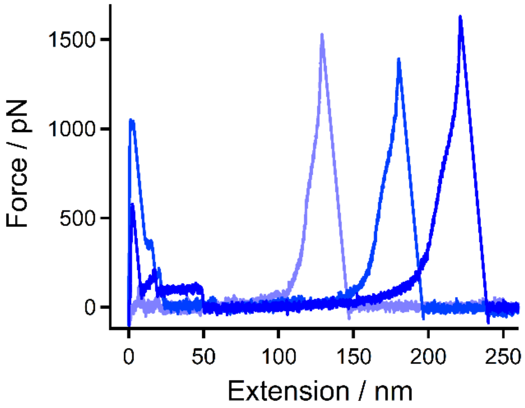

Figure S4 Typical original F-E curves of chitin obtained in pH=9.

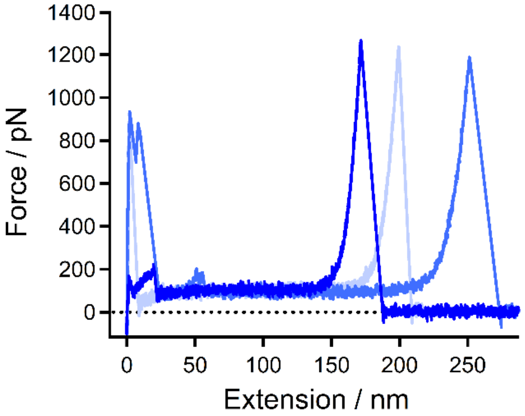

Figure S5 Typical original F-E curves of chitin obtained in pH=11.

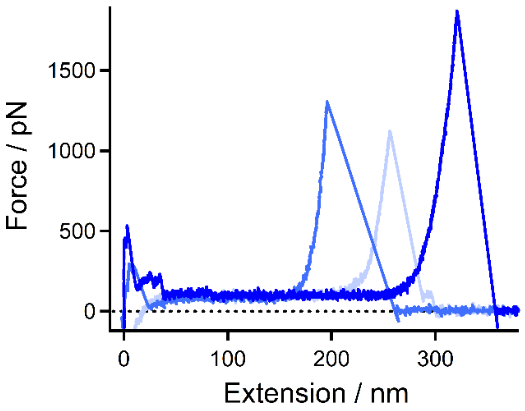

Figure S6 Typical original F-E curves of chitin obtained in pH=13.

It has been reported that FRC model is suitable to describe the elasticity of flexible chains(such as C-C and S-S backbone) [1], FJC model is widely applicated for polymers that the backbone contains ring (such as polysaccharides DNA and RNA) , which are rather rigid [2]. WLC model is suitable for more rigid polymers such as proteins and multi-row covalently bonded molecules. In this study, both TSQM-WLC and TSQM-FRC models were failed to describe the elasticity of chitin (Figure S7).

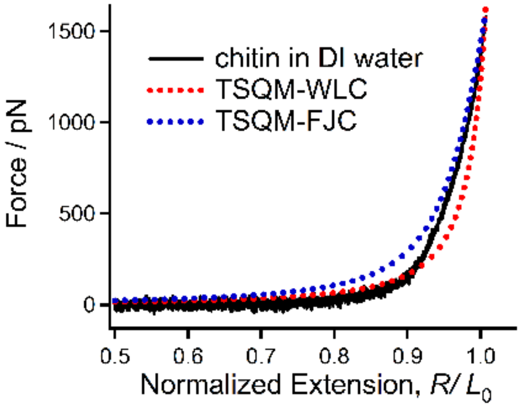

Figure S7. Fitting results of TSQM-FJC and TSQM-FRC models on the F-E curves of chitin obtained in DI water. Obvious deviations can be noticed between the theoretical curves and the experiment curve.

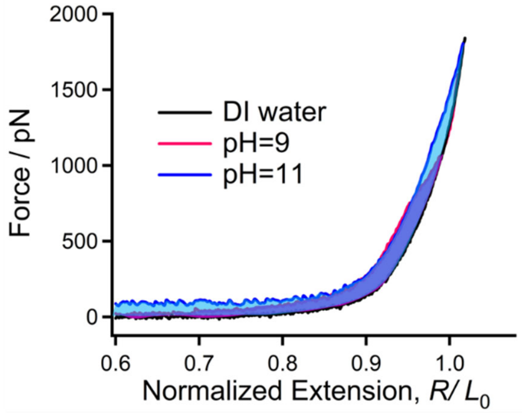

Figure S8. The area difference of chitin in alkaline solutions compared to in DI water.

The ΔG between pH=9 and DI water is calculated to be 12.95 kJ/(mol·unit)), which is about 65% of the ΔG between pH=11 and DI water (19.98 kJ/(mol·unit)). Therefore, the degree of chitin to collapse into a hydrophobic globule in pH=9 is about 65% compared to the final state in alkaline solutions (pH≥11) (Figure S8).

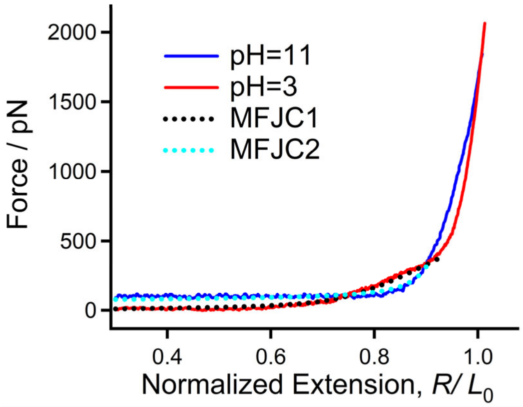

Figure S9. MFJC model fitting on the F-E curves of chitin in pH=3 and 11.

The elasticity of a polymer chain can be reflected by its elastic modulus, which can be precisely obtained by MFJC model fitting on the experimental F-E curves. As shown in Figure S9, the typical F-E curve of chitin obtained in pH=3 is superposed well with the fitting curves at the low forces. Note that the elastic modulus of a single chitin chain in pH=3 (2.185 nN) is much lower than that in pH=12 (14.175 nN). Since the larger elastic modulus corresponds to the poor elasticity, chitin shows considerable molecular elasticity in acid rather than in alkaline (Figure S9).

The end-end distance of a molecular chain is a classic parameter to directly reflect the conformational behavior, a larger end-end distance value corresponds to a more spreading conformation. The end-end distance of chitin in HCl solution (pH=3), pure water, NaOH solution (pH=11) calculated from the MD simulations is 26.88 ± 0.14 Å, 15.53 ± 0.23 Å and 45.79 ± 0.12 Å respectively. The end-end distance of chitin udner pH=3 was normalized to 1 for direct comparison. The relative value for pure water and pH=11 is 0.59 and 0.34 respectively. The result indicates directly that the degree of expansion of chitin under pH=11 is only 34% of that under pH=3.

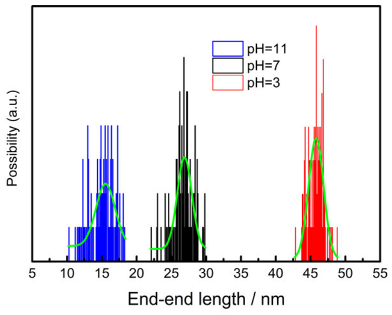

Figure S10. Statistical result of the end-end distance of chitin in pH=3, pure water and pH=11.

Supporting references

1. Zhang, F.; Gong, Z.; Cai, W.; Qian, H.-j.; Lu, Z.-y.; Cui, S., Single-chain mechanics of cis-1, 4-polyisoprene and polysulfide. *Polymer* **2022**, 240, 124473.
2. Bao, Y.; Luo, Z.; Cui, S., Environment-dependent single-chain mechanics of synthetic polymers and biomacromolecules by atomic force microscopy-based single-molecule force spectroscopy and the implications for advanced polymer materials. *Chem. Soc. Rev.* **2020,** 49, 2799-2827.
